# Comparison of Postocclusion Pressure Surge Between Pressure Sensing and Traditional Phacoemulsification Handpieces

**DOI:** 10.1155/joph/3264880

**Published:** 2025-10-16

**Authors:** Tyler Whitaker, Tanner K. Nelson, Reiker G. Ricks, Kolja Klug, Ivan A. Cardenas, Randall J. Olson, Jeff H. Pettey

**Affiliations:** ^1^Department of Ophthalmology and Visual Sciences/John A. Moran Eye Center, University of Utah, Salt Lake City, Utah, USA; ^2^School of Medicine, University of Utah, Salt Lake City, Utah, USA; ^3^Department of Engineering/Center for Medical Innovation, University of Utah, Salt Lake City, Utah, USA

## Abstract

**Purpose:**

To compare the Active Sentry and OZil handpieces regarding their ability to prevent postocclusion surge.

**Setting:**

John A. Moran Eye Center, University of Utah, Salt Lake City, Utah.

**Design:**

Experimental study.

**Methods:**

This study used the Alcon Centurion surgical platform, with both OZil handpieces fitted with balanced tips. The quad preset was used with a vacuum of 500 mmHg, an aspiration flow of 50 mmHg, and an intraocular pressure (IOP) of 70 mmHg. A rubber disk was fixed within a sealed chamber fitted with an electric pressure sensor to monitor pressure changes. The phaco tip was inserted through a small opening, and the foot pedal was set to position two. The tip was put in contact with the fixed rubber disk to replicate tip occlusion and then pulled from the disk to simulate occlusion break. 10 trials were performed with each handpiece, and pressure changes were recorded electronically.

**Results:**

A significant difference (*p* < 0.01) was found between the magnitude of postocclusion surge, as well as between the duration of postocclusion surge.

**Conclusion:**

The Active Sentry handpiece was found to have a decreased magnitude and shorter duration of postocclusion surge compared with the OZil, signifying that having the pressure sensor in the handpiece allows it to react to post-occlusion surge more quickly and to decrease surge magnitude.

## 1. Introduction

One of the main drivers of innovation of phacoemulsification (phaco) technology is the desire to reduce posterior capsular rent (PCR) complications. PCR is the most common potentially sight-threatening intraoperative complication during cataract surgery [1, 2]. A common cause of PCR is contact of the posterior capsule with the phaco tip following a rapid change in intraocular pressure (IOP), which is termed postocclusion surge. Postocclusion surge is defined as a drop in IOP that occurs after an occlusion of the tip clears, most often due to a fragment of lens in the tip [3, 4]. This negative pressure surge relative to the set IOP occurs because while the tip is occluded, vacuum pressure builds, which causes compression of the outflow tubing. When the lens fragment is cleared from the tip, the outflow tubing reverts to its original shape, drawing fluid out of the eye and causing a negative pressure surge. When fluid is drawn out of the eye during surge, the posterior capsule can be drawn towards the phaco tip, with the fluid escaping the eye. If the posterior capsule contacts the tip, a tear can form in the posterior capsule [5].

Studies have shown that it is possible to mitigate surge-induced PCR risk by operating at a high IOP and decreasing the vacuum limit [6]. The drawback is the potential for reduced corneal clarity and/or anterior chamber inflammation [7, 8]. Consequently, there is incentive to mitigate surge effectively, even when operating at a lower IOP. A previous study has shown that the newly developed Active Sentry handpiece, developed by Alcon, clinically improves surge [9].

The Active Sentry technology was developed to maintain tighter control of intraoperative IOP, thereby reducing the risk of PCR due to post-occlusion surge. The Active Sentry handpiece differs from its predecessor, the traditional OZil handpiece, in that it has its pressure sensor in the handpiece itself instead of being located within the machine. Having the pressor sensor in the handpiece may allow pressure changes in the anterior chamber to be sensed and reacted to more rapidly, due to increased proximity of the sensor to the pressure changes being monitored. In this initial proof-of-concept study, a test chamber capable of monitoring pressure fluctuations in real time was developed to measure the Active Sentry's control of post-occlusion surge compared to a traditional phaco handpiece.

## 2. Materials and Methods

This laboratory study used no animal or human subjects and required no Institutional Review Board or Animal Care Committee ethics approval.

### 2.1. Pressure Chamber

**Figure 1:**
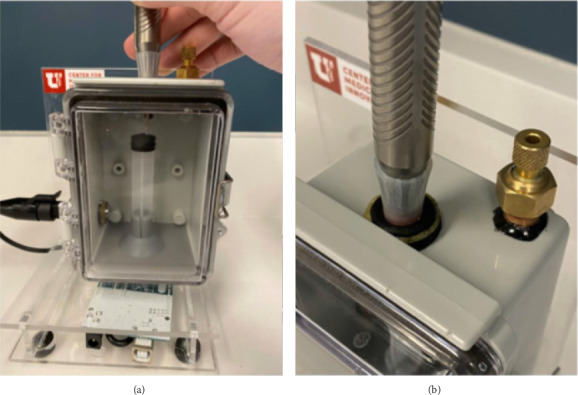
(a) and (b) Chamber used for simulating the intraocular environment and measuring the pressure changes.

A water-tight, rigid chamber was developed to measure the pressure changes within it (Figures 1(a) and 1(b)). A water-tight port was placed in the top of the chamber to introduce the phaco handpiece into the chamber. A rubber sleeve was fitted around the handpiece to ensure a water-tight seal. A rubber stopper mounted within the chamber as an object was used to simulate occlusion by a lens fragment. A bleeder valve was placed in the top of the chamber to remove all excess air from the system. The chamber was fitted with an electronic pressure sensor to record changes in pressure during the trials. The sensor was coded to record 10 data points per second.

### 2.2. Postocclusion Surge Testing

Trials were run using the following settings: vacuum of 500 mmHg, aspiration flow of 50 mL/min, and IOP of 70 mmHg. Settings were selected as they are within the commonly used levels for chop and quadrant steps of surgery, where surge events are most likely to occur. All trials were run with the foot pedal depressed to position 2 for the entirety of the trial.

For each trial, the handpiece was introduced into the chamber and sealed. The bleeder valve was opened, and the foot pedal was depressed to position 1 to fill the chamber and remove any excess air. The quality of the seal was also assessed at this time to ensure that no fluid was leaking from the port where the handpiece entered the chamber.

An investigator used a timer for each trial. At the starting mark, the foot pedal was depressed to position 2 and given 7 s to pressurize to the prespecified IOP. At the 7 s mark, the handpiece was maneuvered to occlude on the rubber stopper mounted within the chamber. At the 14 s mark, occlusion was broken, and data recording continued to measure the pressure changes postocclusion for another 7 s.

### 2.3. Data Analysis

Microsoft Excel was used for data compilation. The first author created the methodology for study data analysis.

To calculate the magnitude and duration of the postocclusion surge, it was necessary to mathematically define the start and stop of postocclusion surge in each trial so that we could reliably measure the duration and magnitude of the surge. We defined the beginning of the postocclusion surge as when the slope of the line between adjacent data points on the curve of the pressure tracing for a given trial was more negative than −6.00 mgHg/s. We chose a slope of −6.00 mmHg/s as a cut off because we observed variation of up to 0.51 mmHg per 10th of a second in our data that we found to be attributed to the resolution of the sensor which we had funding to purchase. Pressure variations that had a calculated slope of −6.00 mmHg/s between data points were always indicative that postocclusion surge was occurring and that the fluctuation was not secondary to the sensitivity of the chamber's pressure sensor.

We developed a nomenclature to define the chronology of each pressure tracing segment. Segment A-to-B represents the rise in pressure when the foot pedal was depressed to position two. The pressure in the chamber when the handpiece was inserted into the surge chamber was not equal to the set IOP. Consequently, a period of time was needed to pressurize the chamber to the set IOP. Segment B-to-C represents the period of occlusion. Segment C-to-D is the postocclusion surge segment with a period of negative relative pressure. Segment D-to-E is unoccluded equilibrium when the foot pedal remained in position 2.

The end of the postocclusion surge was the moment when the slope of the pressure tracing inflected from negative to positive, indicating that the phaco machine had sensed the pressure drop and had begun to react. The pressure at the given time point when surge was defined as beginning or ending was the pressure used to calculate the magnitude of the surge for each trial. Statistical significance was defined as *p* ≤ 0.01. We used the Student's *T*-test to demonstrate significance.

## 3. Results

**Figure 2:**
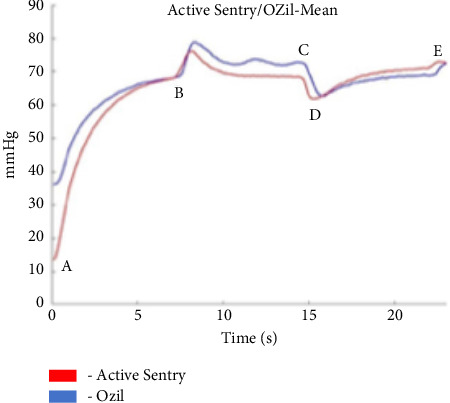
Pressure tracing showing the mean pressures from each trial for the Active Sentry and OZil handpieces.

**Figure 3:**
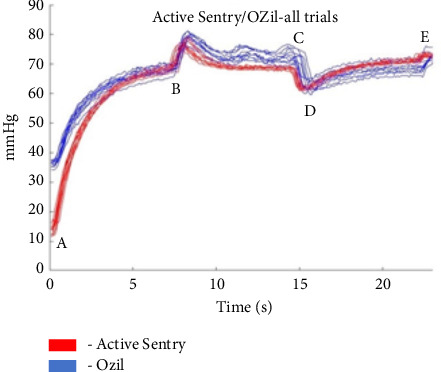
Pressure tracings for each trial for the Active Sentry and OZil handpieces.

A statistically significant difference (*p* < 0.0001) in both magnitude and duration of postocclusion surge was found between the Active Sentry handpiece and the OZil handpiece (Figures 2 and 3). The mean magnitude of surge for the Active Sentry and OZil handpieces was 5.06 mmHg ± 0.92 versus 9.25 mmHg ± 1.85, respectively. The mean duration of surge for the Active Sentry and OZil handpieces was 0.36 s ± 0.07 versus 0.59 s ± 0.11, respectively.

## 4. Discussion

Using our surge monitoring chamber, we were able to compare the magnitude and duration of postocclusion surge between a traditional phaco handpiece, which has a pressure sensor in the phaco unit, and the Active Sentry, which has its pressure sensor in the handpiece itself. We hypothesized that having a pressure sensor in the handpiece would allow the phaco system to sense surge sooner and react more quickly than it would if it did not include a handpiece pressure sensor. Our model validated this hypothesis. We found that the magnitude of the postocclusion surge was 55% less with the Active Sentry handpiece than with the OZil handpiece. Furthermore, we found that the duration of postocclusion surge was 61% shorter with the Active Sentry handpiece than the traditional handpiece. These significant findings quantitatively support that having a pressure sensor located within a phaco handpiece appreciably reduces postocclusion surge. However, it is difficult to apply these quantitative measurements to the clinical setting. Knowing that there is a relative decrease in surge with a pressure sensor in the handpiece, it would be reasonable to expect significantly less surge in a clinical setting.

Our experiment also proves that postocclusion surge can be measured in this manner. This was the initial evaluation of our pressure surge monitoring chamber, designed in conjunction with the University of Utah Center for Medical Innovation. To the best of our knowledge, our approach to measuring postocclusion surge differs from any previously published papers evaluating surge [3, 6, 8, 10–12]. Recent bench studies have used compliant chambers that measure volumetric changes within the chamber to measure postocclusion surge, stating that they believe volumetric changes to be more clinically relevant [6, 12]. While we believe both models are valid ways to quantify surge given the mathematical relationship between pressure and volume, our pressure-sensing chamber offers a way to look at surge in the same units in which IOP is measured and to allow visualization of what the pressure tracings look like for various phaco technologies. Other studies evaluating postocclusion surge have been performed intraoperatively in human and rabbit eye studies [13, 14]. These studies have the surgeon place a pressure probe through a small paracentesis to measure intraoperative pressure changes within the eye. While these studies are incredibly insightful as well, our model excels at reducing variables that inherently accompany live models during an actual cataract extraction. Our model was never meant to replicate the intraocular environment. It was meant to create a simplified environment that would allow us to reliably determine how quickly a phaco device would be able to sense and correct postocclusion surge.

A question might be raised regarding the fact that the pressure reading at time equals 0 seconds on our graphs are not equal. We noticed this as well during data collection. We hypothesize that the reason for this is that there was variability in the force applied when inserting the handpiece into the chamber to create the water-tight seal, causing a variable starting pressure. For this reason we built a pressurization period into our experiment (segment A-B) in order to assure that no matter what the variability of the starting pressure was, the pressure at *t* = 7 s would be equivalent prior to creating occlusion. Adding this pressurization period to the beginning of the experiment allowed us to have replicable trials that produced valid results.

As with all experiments, there are limitations and potential sources of error. We present an in vitro model for quantifying pressure changes resulting from varying phaco technologies [15]. It is not an in vivo, or tissue surrogate simulator, as ours is a rigid and noncompliant model. Funding issues prevented us from constructing the more complex chambers that others have used for surge measurement [6, 12, 16, 17]. Nonetheless, we feel that the pressure sensor that we constructed succeeds at creating a replicable environment in which we could observe pressure tracings and directly compare various aspects of phaco technologies.

One source of error we identified early in the development of our model was the effect on residual air in our system. As air is compressible, residual air within the chamber will dampen pressure changes sensed by the built-in sensor. To minimize this source of error, a valve was incorporated in at the uppermost portion of the chamber to bleed excess air within the chamber. We observed that wiping down the chamber with alcohol swabs prevented small bubbles from sticking to the walls of the chamber as well.

Additionally, pressure measurements in the system could be affected when the operator moves the handpiece into position. To minimize this effect, our model required only horizontal motions to occlude and unocclude the phaco tip, thereby limiting movements with direct vectors into the system. Furthermore, we used the same operator for every experiment to minimize inter-user variability.

During this experiment, we observed the surge-related implications of moving the pressure sensor adjacent to the eye rather than the delayed measurement conducted over the course of the tubing. We look forward to using this model to evaluate other factors affecting postocclusion surge. In future studies we plan to manipulate the parameters to evaluate changes across the spectrum of settings that a typical cataract surgeon may use. Understanding that this is still a source for the introduction of error to our experiment, our team has since updated the chamber to be able to create occlusion and break it without moving the handpiece at all, using a corkscrew mechanism within the chamber to allow the stopper used to create occlusion to move to the phaco tip as opposed to the other way around.

## Data Availability

The data that support the findings of this study are available from the first author, Tyler Whitaker (tyler.whitaker@hsc.utah.edu), upon reasonable request.
